# The dataset of the effects of fly ash on growth and yield of radish plant under two types of soil

**DOI:** 10.1016/j.dib.2024.110234

**Published:** 2024-02-26

**Authors:** Van Loc Nguyen, Duc Huy Nguyen

**Affiliations:** a Faculty of Agronomy, Vietnam National University of Agriculture, Hanoi, Viet Nam; b Plant Clinic, Vietnam National University of Agriculture, Viet Nam

**Keywords:** Radish plant, Fly ash, Delta clay rich soil, Coastal sandy soil, Storage-root

## Abstract

This article presents data of the effects of fly ash on growth and yield of radish plant under two types of soil (delta clay rich soil and coastal sandy soil). The experiment was conducted under semi-controlled conditions in a greenhouse at the Faculty of Agronomy, Vietnam National University of Agriculture (latitude 21°0′01N, longitude 105° 9′32″ W). The experiment has been conducted with the Randomized Complete Block Design (RCBD), each experimental formula was repeated 5 times. A total of 10 experimental formulas were performed including 100% delta clay rich soil, 95% delta clay rich soil+5% FA, 90% delta clay rich soil+10% FA, 85% delta clay rich soil+15% FA, 80% delta clay rich soil+20% FA, 100% coastal sandy soil, 95% coastal sandy soil+5%FA, 90% coastal sandy soil +10%FA, 85% coastal sandy soil+15%FA, and 80% coastal sandy soil+20%FA. Data on germination rate, plant height, number of leaves, SPAD values, leaf area, shoot fresh and dry weight, storage-root traits, storage-root fresh and dry weight were collected to assess the effects of fly ash on growth and yield of radish plant under delta clay rich soil and coastal sandy soil. This data could help develop a strategy fly ash application for crop cultivation.

Specifications Table

**Table:** 

Subject	Agricultural Science
Specific subject area	Agronomy
Data format	Raw
Type of data	Tables
How data were acquired	We used a two-way analysis of variance (ANOVA) to compare the average values among treatments. Least significant difference (LSD) test was used to find the means that were significantly different from each other. Data were analyzed using the IRRISTAT 5.0 software.
Data format	Raw
Data collection	The experiment was conducted under semi-controlled conditions in a greenhouse. A total of 10 experimental formulas were performed including 100% delta clay rich soil, 95% delta clay rich soil+5% FA, 90% delta clay rich soil+10% FA, 85% delta clay rich soil+15% FA, 80% delta clay rich soil+20% FA, 100% coastal sandy soil, 95% coastal sandy soil+5%FA, 90% coastal sandy soil +10%FA, 85% coastal sandy soil+15%FA, and 80% coastal sandy soil+20%FA. The germination rate was recorded at 2nd, 3rd, 4th, 5th, 6th and 7th after sowing. Plant height and number of leaves were measured at 11st, 21st, 31st, 41st, 51st, 61st and 71st after sowing. SPAD values were collected at 30th, 42nd, 54th, 66th after sowing. The leaf area, shoot fresh and dry weight, storage-root traits, storage-root fresh and dry weight measured at the harvest stage. Plant height is the height from the tip of the longest leaf. SPAD index using SPAD (Konica-Minolta 502, Japan). Leaf area was calculated by leaf area meter (Li-3100, Lin Coln Nebraska USA). Fresh weight was measured by using electronic balance (OHAUS PR4202, USA). Dry weight of tuber was determined after drying samples at 80 °C for three days in drying chamber (BINDER, USA) until constant weight. Dry samples were weighted by the electronic balance (OHAUS STX-223, USA).
Data source location	Raw data was stored in OSFHOME
Data accessibility	Repository name: OSFHOME Data identification number: ezck8 Direct URL to data: https://osf.io/ezck8

## 1 Value of the Data

• The dataset illustrates the effects of fly ash on growth and yield of radish plant under two types of soil (delta clay rich soil and coastal sandy soil)..
• The data could be valuable for researchers studying on fly ash application for crops.
• The data also provides a strategy for using fly ash for radish cultivatin under different soil types. It normally had positive effect to the radish in 5% and 10% fly ash.

## 2 Background

Fly ash (FA) has been well-described as a combustible brownish-black sedimentary rock that has been used as a crucial energy source for industries and power generation around the world. However, the burning of coal has been identified as one of the major causes of global environmental issues such as air pollution, climate change, and environmental degradation. Recently, FA has been shown to have potential applications in agriculture, particularly in soil amendment and crop production. However, FA for crops under soil types remains unclear.

## 3 Data Description

Table 1 presents data on effects of fly ash the germination rate (%) of radish plant under two types of soil . The raw data for Table 1 is presented in the Supplementary file “Germination rate”. Data on plant height (Table 2), number of leaves (Table 3), SPAD values (Table 4), leaf area (Table 5), shoot fresh and dry weight (Table 6), storage-root traits (Table 7), storage-root fresh and dry weight (Table 8) were collected to assess the effects of fly ash applicaton rate, soil types and their interaction on the growth and yield of radish plant. The raw data for Tables 2, 3, 4, 5, 6, 7 and 8 is presented in the Supplementary file “Plant height”, “Number of leaves”, “SPAD value”, “Leaf area”, “Fresh and dry weight of shoot”, “Storage root traits”, and “Root fresh and dry weight ”, respectively.

**Table 1:** Effects of fly ash (FA) application on germination rate (%) of radish plant under two types of soil (S).

Formula		Germination rate (%) at …days after sowing					
Soil type	FA rate	2	3	4	5	6	7
	0%	8	56	84	96	96	100
	5%	28	80	92	100	100	100
**Delta clay rich soil**	10%	40	76	84	92	100	100
	15%	4	44	76	96	100	100
	20%	24	80	88	92	100	100

*Average*		20.8	67.2	84.8	96	99.2	100

	0%	92	100	100	100	100	100
	5%	76	100	100	100	100	100
**Coastal sandy soil**	10%	64	96	100	100	100	100
	15%	84	100	100	100	100	100
	20%	100	100	100	100	100	100

*Average*		83.2	99.2	100	100	100	100

**Table 2:** Effects of fly ash (FA) application on plant height (cm) of radish plant under two types of soil (S).

Formula		Plant height of radish at…days after sowing						
Soil type	FA rate	11	21	31	41	51	61	71
	0%	8.32	11.26	13.18	17.22	25.96	28.2	29.72^a^
	5%	9.02	14.12	16.42	20.88	27.42	29.3	30.74^a^
**Delta clay rich soil**	10%	10.1	14.18	16.76	20.72	27	29.16	30.58^a^
	15%	9.14	13.22	14.78	18.1	26.16	28.7	30.12^a^
	20%	8.66	13.1	14.76	16.82	25.6	28.2	29.98^a^

*Average*		9.05	13.18	15.18	18.75	26.43	28.71	30.23

	0%	9.02	10.22	12.96	14.36	17.58	19.08	20.04^c^
	5%	9.38	13.2	14.68	16.94	19.64	21.6	23.64^b^
**Coastal sandy soil**	10%	9.16	13.62	15.06	17.22	20.14	22	24.94^b^
	15%	8.74	13.6	14.78	17.14	19.6	21.9	24.62^b^
	20%	7.86	10.12	11.7	13.3	18.2	21.5	24.6^b^

*Average*		8.83	12.15	13.84	15.79	19.03	21.22	23.57

*LSD_0.05(S)_*		0.20	0.29	0.35	0.43	0.56	0.67	0.70
*LSD_0.05(FA)_*		0.32	0.47	0.56	0.69	0.88	1.07	1.11
LSD_0.05(S*FA)_		0.45	0.66	0.79	0.97	1.25	1.51	1.56

Each value is the mean of five replicates. Different letters within the same column indicate the least significant difference values at the 0.05 level (LSD_0.05__S*FA_) for the different soil types at the defefferent FA rates.

**Table 3:** Effects of fly ash (FA) application on number of leaves of radish plant under two types of soil (S).

Formula		Number of leaves at … days after sowing						
Soil type	FA rate	11	21	31	41	51	61	71
	0%	2	5	9	10.4	14.4	20.2	17.8^bc^
	5%	2	5	9	11	13.5	16.8	19.4^a^
**Delta clay rich soil**	10%	2	5	8.8	11.4	14.2	18.4	19.4^a^
	15%	2	4	8	10.4	13.4	18.6	18.4^b^
	20%	2	3.2	7	9.2	13.6	16.5	17.4^c^

*Average*		2	4.44	8.36	10.48	13.82	18.1	18.48

	0%	3	4	7.2	10.4	11.4	13.6	12.4^g^
	5%	3	3.8	6.8	8.8	10.75	14.8	15.6^d^
**Coastal sandy soil**	10%	2.6	3.6	7.8	9.2	10.6	14.6	14.5^e^
	15%	2.8	3.8	7.2	9	10.6	15	15.4^d^
	20%	3	4	6.6	8	9.4	12.5	13.6^f^

*Average*		2.88	3.84	7.12	9.08	10.55	14.1	14.3

*LSD_0.05(S)_*		0.15	0.17	0.21	0.27	0.31	0.32	0.29
*LSD_0.05(FA)_*		0.24	0.27	0.33	0.42	0.49	0.51	0.46
LSD_0.05(S*FA)_		0.34	0.39	0.46	0.59	0.69	0.72	0.64

Each value is the mean of five replicates. Different letters within the same column indicate the least significant difference values at the 0.05 level (LSD_0.05__S*FA_) for the different soil types at the defefferent FA rates.

**Table 4:** Effects of fly ash (FA) application on SPAD value of radish plant under two types of soil (S).

Formula		SPAD values of radish plant at …days after sowing			
Soil type	FA rate	30	42	54	66
	0%	33.2	37.9	47.02	52.96^a^
	5%	35.48	38.08	46.82	52.44^a^
**Delta clay rich soil**	10%	32.56	37.14	45.44	49.74^b^
	15%	34.68	40	46.94	48.8^bc^
	20%	34.96	39.1	41.96	48.18 ^bc^

*Average*		34.176	38.444	45.636	50.424

	0%	33.02	37.54	47.86	48.34 ^bc^
	5%	33.32	38.50	44.28	47.1^c^
**Coastal sandy soil**	10%	35.14	38.76	44.12	48.04 ^bc^
	15%	35.94	37.28	44.58	44.7 ^d^
	20%	32.96	36.18	43.34	43.5 ^e^

*Average*		34.076	37.650	44.836	46.336

*LSD_0.05(S)_*		0.666	0.733	0.692	0.939
*LSD_0.05(FA)_*		1.052	1.159	1.094	1.484
LSD_0.05(S*FA)_		1.488	1.639	1.547	2.099

Each value is the mean of five replicates. Different letters within the same column indicate the least significant difference values at the 0.05 level (LSD_0.05__S*FA_) for the different soil types at the defefferent FA rates.

**Table 5:** Effects of fly ash (FA) application on leaf area (dm^2^ plant^−1^) of radish plant under two types of soil (S).

Formula		Leaf area (dm^2^ plant^−1^)
Soil type	FA rate	
	0%	16.873^b^
	5%	19.774^a^
**Delta clay rich soil**	10%	16.894^b^
	15%	16.557^c^
	20%	16.094^d^

*Average*		17.24

	0% (Sample2)	10.773^g^
	5%	10.594^gh^
**Coastal sandy soil**	10%	12.195^f^
	15%	12.791^e^
	20%	10.366^h^

*Average*		13.24

*LSD_0.05(S)_*		0.371
*LSD_0.05(FA)_*		0.586
LSD_0.05(S*FA)_		2.616

Each value is the mean of five replicates. Different letters within the same column indicate the least significant difference values at the 0.05 level (LSD_0.05__S*FA_) for the different soil types at the defefferent FA rates.

**Table 6:** Effects of fly ash (FA) application on shoot fresh and dry weight (g plant^−1^) of radish plant under two types of soil (S).

Formula		Shoot fresh weight (g plant^−1^)	Shoot dry weight (g plant^−1^)
Soil type	FA rate		
	0%	78.320^b^	5.698^b^
	5%	92.326^a^	7.058^a^
**Delta clay rich soil**	10%	79.832^b^	6.472^ab^
	15%	71.662^c^	6.119^b^
	20%	69.974^c^	5.823^b^

*Average*		78.42	6.23

	0%	31.012^e^	2.976^c^
	5%	32.996^e^	3.237^c^
**Coastal sandy soil**	10%	37.454^d^	3.55^c^
	15%	38.886^d^	3.737^c^
	20%	32.762^e^	3.224^c^

*Average*		34.62	3.34

*LSD_0.05(S)_*		1.17	0.118
*LSD_0.05(FA)_*		1.85	0.187
LSD_0.05(S*FA)_		2.616	0.25

Each value is the mean of five replicates. Different letters within the same column indicate the least significant difference values at the 0.05 level (LSD_0.05__S*FA_) for the different soil types at the defefferent FA rates.

**Table 7:** Effects of fly ash application on storage-root diameter (cm) and storage-root length (cm) of radish plant under two types of soil.

Formula		Storage-root diameter (cm)	Storage-root length (cm)
Soil type	FA rate		
	0%	3.03^bc^	12.66^d^
	5%	3.15^ab^	16.66^a^
**Delta clay rich soil**	10%	3.29^a^	17.36^a^
	15%	2.95^c^	14.90^b^
	20%	2.74^d^	14.06^c^

*Average*		3.03	15.13

	0%	2.15^f^	10.24^e^
	5%	2.95^c^	12.32^d^
**Coastal sandy soil**	10%	2.51^e^	13.70^c^
	15%	2.50^e^	12.16^d^
	20%	2.16^f^	10.46^e^

*Average*		2.45	11.78

*LSD_0.05(S)_*		0.719	0.326
*LSD_0.05(FA)_*		0.14	0.515
LSD_0.05(S*FA)_		0.161	0.729

Each value is the mean of five replicates. Different letters within the same column indicate the least significant difference values at the 0.05 level (LSD_0.05_) for the different treatments.

**Table 8:** Effects of fly ash (FA) application on storage-root fresh and dry weight (g plant^−1^) of radish plant under two types of soil (S).

Formula		Storage-root fresh weight (g plant^−1^)	Storage-root dry weight (g plant^−1^)
Soil type	FA rate		
	0%	64.33^c^	4.41^c^
	5%	84.91^b^	5.99^a^
**Delta clay rich soil**	10%	110.56^a^	9.17^a^
	15%	65.91^c^	4.58^c^
	20%	57.91^d^	3.59^d^

*Average*		76.72	5.55

	0%	17.73^h^	1.62^g^
	5%	57.44^d^	3.35^e^
**Coastal sandy soil**	10%	45.78^e^	3.50^ed^
	15%	40.17^f^	2.73^f^
	20%	22.07^g^	1.80^g^

*Average*		36.64	2.60

*LSD_0.05(S)_*		1.564	0.112
*LSD_0.05(FA)_*		2.473	0.177
LSD_0.05(S*FA)_		3.498	0.25

Each value is the mean of five replicates. Different letters within the same column indicate the least significant difference values at the 0.05 level (LSD_0.05_) for the different treatments.

## 4 Experimental Design, Materials and Methods

### 4.1 Materials

*Radish:* White radish variety F1 LONG WHITE is a French radish variety that grows and develops well, has high disease resistance, spoon-shaped leaves, short leaves, and bright green.

*Delta clay rich soil:* The delta clay-rich soil was taken at the field of Hanoi (latitude 21°0′01N, longitude 105° 9′32″ W). This soil is composed of 45% clay, 1.72% organic matter; 6.0 pH and 23.6 meq/100 g CEC [1].

*Coastal sandy soil:* Coastal sandy soil was taken from a costal farmland at a depth of 0–30 cm (latitude 17^0^14′00’’and longtitude 106 49′ 00′' East). The characteristics of soil are composed of 97% sandy, 0.18 organic matter, 7.0 pH, and 4.29 meq/100 g CEC [1].

*Fly ash (FA):* The fly ash recovered from the Mong Duong 2 coal-fired thermal power plant and had the main chemical composition of oxygen (43%), Si (26%), Al (15%), K (9%), and Fe (6%) [2].

### 4.2 Experimental design and treatments

The experiment was conducted under semi-controlled conditions in a greenhouse at the Faculty of Agronomy, Vietnam National University of Agriculture (latitude 21°0′01N, longitude 105° 9′32″ W). Five kilogrammes of all treatment mixtures were placed into plastic pots (30 × 15 × 20 cm). The experiment has been conducted with the Randomized Complete Block Design (RCBD), each experimental formula was repeated five times. The FA application was used according to Singh et al. [3] as A total of 10 experimental formulas were performed including 100% delta clay rich soil, 95% delta clay rich soil+5% FA, 90% delta clay rich soil+10% FA, 85% delta clay rich soil+15% FA, 80% delta clay rich soil+20% FA, 100% coastal sandy soil, 95% coastal sandy soil+5%FA, 90% coastal sandy soil +10%FA, 85% coastal sandy soil+15%FA, and 80% coastal sandy soil+20%FA. Each experimental pot was supplied fertilizers of 0.81gN, 0.54gP_2_O_5_ and 0.54gK_2_O. Seeds of radish were soaked in fresh water in 3 h. Then five seeds were sown in each pot. At 4 leaves stage, plants were thined to one plant per pot.

### 4.3 Measurements

The germination rate was recorded at 2nd, 3rd, 4th, 5th, 6th and 7th after sowing. Plant height and number of leaves were measured at 11st, 21st, 31st, 41st, 51st, 61st and 71st after sowing. SPAD values were collected at 30th, 42nd, 54th, 66th after sowing. The leaf area, shoot fresh and dry weight, storage-root traits, storage-root fresh and dry weight measured at the harvest stage. Plant height is the height from the tip of the longest leaf. SPAD index using SPAD (Konica-Minolta 502, Japan). Leaf area was calculated by leaf area meter (Li-3100, Lin Coln Nebraska USA). Fresh weight was measured by using electronic balance (OHAUS PR4202, USA). Dry weight of tuber was determined after drying samples at 80 °C for three days in drying chamber (BINDER, USA) until constant weight. Dry samples were weighted by the electronic balance (OHAUS STX-223, USA).

### 4.4 Data analysis

We used a two-way analysis of variance (ANOVA) to compare the average values among treatments. Least significant difference (LSD) test was used to find the means that were significantly different from each other. Data were analyzed using the IRRISTAT 5.0 software.

## Limitations

Not applicable.

## Ethics Statement

The authors declare that they confirm that the authors have read and follow the ethical requirements for publication in Data in Brief and confirming that the current work does not involve human subjects, animal experiments, or any data collected from social media platforms.

## CRediT authorship contribution statement

**Van Loc Nguyen:** Conceptualization, Methodology, Software, Data curation, Writing – original draft. **Duc Huy Nguyen:** Visualization, Investigation, Software, Validation, Writing – review & editing.

## Data Availability

The dataset of the effects of fly ash application on the growth and yield of Radish plant (Original data) (OFS HOME).

## Appendix Supplementary materials

Image, application 1
